# Fabry–Perot Cavity Optimization for Absolute Strain Sensing Using Finite Element Analysis

**DOI:** 10.3390/s23218785

**Published:** 2023-10-28

**Authors:** João M. B. Pereira, Paula M. P. Gouvea, Arthur M. B. Braga, Isabel C. S. Carvalho, Antonio C. Bruno

**Affiliations:** 1 Department of Physics, PUC-Rio, Rua Marquês de São Vicente 225, Gavea, Rio de Janeiro 22451-900, Brazil; isabel.carvalho@puc-rio.br (I.C.S.C.); acobruno@gmail.com (A.C.B.); 2 Research Institute of Sweden (RISE), Fiber Optics, Isafjordsgatan 22, 16440 Kista, Sweden; 3 Optical Fiber Sensors Lab (LSFO), PUC-Rio, Rua Marquês de São Vicente 225, Gavea, Rio de Janeiro 22451-900, Brazil; pgouvea@puc-rio.br (P.M.P.G.); abraga@puc-rio.br (A.M.B.B.)

**Keywords:** Fabry–Perot interferometer, in-fiber Fabry–Perot, strain sensing, optical fiber sensing, finite element analysis

## Abstract

The finite element method (FEM) was used to investigate the optical–mechanical behavior of a Fabry–Perot Interferometer (FPI) composed of a capillary segment spliced between two sections of standard optical fiber. The developed FEM model was validated by comparing it with theory and with previously published experimental data. The model was then used to show that the absolute strain on the host substrate is usually smaller than the strain measurement obtained with the sensor. Finally, the FEM model was used to propose a cavity geometry that can be produced with repeatability and that yields the correct absolute strain experienced by the host substrate, without requiring previous strain calibration.

## 1. Introduction

Sensors based on fiber-optic Fabry–Perot interferometers (FPIs) have been successfully used to monitor composite materials; civil and electrical engineering structures; and applications in medicine, the aerospace industry, etc. [1]. Some of their key advantages over conventional sensors include immunity to electromagnetic interference and corrosion, small size, low-temperature sensitivity, and high spatial resolution. Some of the most often used techniques for building this type of sensor involve inserting two silica fibers into a glass capillary or splicing standard optical fibers to both sides of a hollow-core fiber, a capillary optical fiber, or a ceramic-derived fiber [2,3,4,5]. Other approaches include the use of PCFs [6,7], femtosecond laser [8,9], or splicing a piece of microfiber to the tip of an SMF [10]. In the literature, optical fiber sensors based on FPIs are also referred to as in-line FPI, in-fiber FPI, air-gap FPI, or FPI cavity.

Different schemes have been proposed for the use of FPI sensors in measuring a variety of physical and chemical quantities such as strain [7,11], pressure [12], magnetic field [4], temperature [13], and humidity [14], among others. Particularly for strain sensing, specific configurations have been proposed with the purpose of increasing the strain sensitivity—defined as the wavelength displacement of the interference spectrum per applied strain (με)—while simultaneously reducing the temperature sensitivity, in order to reduce the temperature-induced error in the strain measurement.

For strain-sensing applications, the fiber containing the Fabry–Perot cavity is usually bonded to the host structure undergoing strain, and it is assumed that the strain undergone by the optical fiber and the FPI cavity is the same as the strain applied to the host structure. However, when longitudinal stress is applied, the mechanical shear deformation of the cavity will occur due to the mechanical characteristics of the optical fiber section containing the cavity and the bonding of the optical fiber to the host structure [9]. Consequently, the strain along the diameter of the optical fiber at the FPI cavity is dependent on the geometry of the cavity and is not necessarily constant along the cavity length, or even the same value as the strain applied to the optical fiber or substrate.

In the present work, the finite element method (FEM) was applied using the COMSOL Multiphysics^®^ 5.3 software to analyze the mechanical and optical responses of an optical fiber Fabry–Perot interferometer (FPI) consisting of a cylindrical air cavity inside an optical fiber. There are few studies using in-fiber FPI and FEM analysis in the literature, such as [15], but unfortunately, the related data are not available for comparison.

COMSOL was preferred for the analysis, as it can conveniently be used for stationary and time-dependent problems with little modifications to the model. The structural mechanic (SM) and electromagnetic wave (EMW) modules were utilized in the model. The relationship between the strain applied to a substrate and the resulting strain applied to an FPI cavity attached to this substrate was determined.

The FEM analysis was first validated by comparing the mechanical–optical behavior and the resulting strain sensitivity of the cavity, initially not bonded to a substrate, to the expected theoretical response of a Fabry–Perot cavity [7,9,16]. For this comparison, a force was applied to elongate the capillary containing the FPI cavity.

Next, a force was applied to a long optical fiber section containing the Fabry–Perot cavity, also not attached to a substrate, and the mechanical deformation, resulting strain, and corresponding wavelength shift were determined. Subsequently, with the purpose of further validating the FEM model, the wavelength shift obtained using the model was compared with the experimental data published in [4].

After validation through the comparison of theoretical and experimental results for an optical fiber not attached to a substrate, the FEM analysis was used to simulate the FPI sensor when bonded to a substrate by means of an adhesive. The strain value obtained using the FPI sensor was compared with the actual strain applied to the substrate, hereafter referred to as the “absolute strain”. The results show that the strain value obtained using the FPI sensor is often higher than the absolute strain actually applied to the substrate. This “amplification” effect is related to the inner diameter of the capillary spliced to the fiber and, ultimately, to the amount of glass composing the glass wall around the cylindrical air cavity. These results indicate that, for most types of cavity geometries reported in the literature, the strain value obtained using the FPI sensor is greater than the absolute strain applied to the substrate, so one should not consider the strain value measured with the sensor as the same as the strain on the substrate. Doing so would result in the incorrect measurement of the absolute strain on the substrate.

This is usually valid in applications where the exact absolute strain value is not necessary, and a proportional value is acceptable. In fact, for measurements of small strain variations, the “amplification” of the measured strain can be an advantage because it acts as an “enhancement” of the strain sensitivity of the sensor, allowing for the monitoring of small strain changes. However, in applications such as the structural health monitoring (SHM) of tunnels, bridges, and aircraft, it is expected and advisable that the FPI sensor yields the correct (absolute) strain undergone by the monitored structure.

Finally, to offer a possible solution for applications where the absolute strain value must be known, the FEM model was used to simulate various FPI cavity proportions (cavity inner diameter X cavity length) to find a specific geometry that yields approximately the same absolute strain to which the substrate was subjected while still maintaining low-temperature sensitivity. This optimized FPI cavity can be an important tool in applications where it is not feasible to perform prior calibration of the FPI/adhesive/substrate system in the laboratory, such as in some of the SHM applications mentioned above.

## 2. Mechanical and Optical Response of an FPI Cavity and Sensor

### 2.1. Theory

The mechanical behavior of an optical fiber of cross-sectional area *A* and Young modulus *E* undergoing a longitudinal force (*F*) can generally be described by

(1) $$\sigma =\frac{F}{A}=E\varepsilon$$

where *σ* is the applied stress, and *ε* is the resulting strain. The strain (*ε*) is the mechanical deformation (Δ*L = L − L*_0_) undergone by the optical fiber divided by its initial length (*L*_0_), where *L* is the final length (*ε =* Δ*L/L*_0_). Therefore, if the optical fiber contains a cavity of initial length *L_CAV_*_0_ and final mechanical deformation Δ*L_CAV_ = L_CAV_ − L_CAV_*_0_; the resulting strain at the cavity will be

(2) $$\varepsilon_{CAV0}=\frac{∆L_{CAV}}{∆L_{CAV0}}$$

when light is guided in the optical fiber, and the cavity acts as a Fabry–Perot interferometer (FPI), the interference spectrum produced at the cavity is related to its geometry. The maximum condition for the interference spectrum is given as follows:

(3) $$m\lambda_{CAV0}=2nL_{CAV0}$$

where *n* is the refractive index of the gas/air inside the cavity, *m* is an integer, and *L_CAV0_* and *λ_CAV_*_0_ are the initial cavity length and the central wavelength at the *m*th interference peak or valley, respectively, when the FPI is not under strain. When the fiber is pulled with a longitudinal force, the cavity will suffer mechanical deformation (elongate), which will shift the interference spectrum produced by it. Using Equation (3), changes in *L_CAV_* or *n* are related as follows:

(4) $$\frac{∆\lambda_{CAV}}{∆\lambda_{CAV0}}=\frac{∆(nL_{CAV})}{nL_{CAV0}}$$

Considering the cavity is usually filled with air (*n* = 1 and Δ*n* = 0), and using Equations (2) and (4), one obtains the relation given by the Fabry–Perot interference theory, where the wavelength shift (Δ*λ_CAV_ = λ_CAV_ − λ_CAV_*_0_) and the change in the cavity length (Δ*L_CAV_*) for a given applied strain (*ε*) are related to each other as follows [7,9,16,17]:

(5) $$\varepsilon_{CAV}=\frac{∆L_{CAV}}{L_{CAV0}}=\frac{∆\lambda_{CAV}}{\lambda_{CAV0}}$$

Equation (5) can be used to calculate the expected theoretical response of an FPI cavity. For example, for an FPI under the strain of 1 με and initial central wavelength of *λ_CAV_*_0_ = 1.5 μm, the theoretical wavelength shift is 1.5 pm.

Another important parameter used for FPI sensors is the strain sensitivity (*S_S_*), which is usually reported as the wavelength shift per applied strain as follows:

(6) $$S_{S}=\frac{{\Delta \lambda }_{CAV}}{\Delta \varepsilon }\;(\mathrm{pm}/\mu \varepsilon )$$

Therefore, for the example above, the theoretical sensitivity response of the cavity is 1.5 pm/με.

Regarding temperature sensitivity, one of the advantages of the in-fiber air cavity, when used as a strain sensor, is that its temperature sensitivity is more than 10 times smaller (typically, 0.8–1.1 pm/°C) than that of a typical fiber Bragg grating (FBG) because it is proportional to the thermal expansion coefficient of glass, as opposed to the thermo-optic effect [4,18].

The following sections will discuss the FEM analysis carried out to model the optomechanical response of the Fabry–Perot cavity in three different cases when a longitudinal force is applied to (a) the cavity, specifically, to the capillary containing the FPI cavity (Section 2.3); (b) an optical fiber section containing the cavity (Section 2.4 and Section 2.5); and (c) the substrate, when the optical fiber is attached to one (Section 3). The analysis allowed for a comparison of the strain at the substrate, the optical fiber, and the cavity, and an investigation of the association between cavity length changes and wavelength shifts in its interference spectrum. The model is based on the equations above, as well as the Poisson ratio (ν), which is used to obtain the perpendicular compression resulting from a longitudinal mechanical deformation.

### 2.2. Finite Element Method Model

The developed FEM model was used to simulate a section of single-mode optical fiber (SMF) containing an FPI cavity, initially with the optical fiber not attached to a substrate, and later with the fiber bonded to the substrate. For the simulations discussed in this work, the SMF was modeled with its cladding diameter, core diameter, and length equal to 125 μm, 8 μm core, and 10 mm, respectively. The cavity was modeled with various lengths and with an inner diameter of 75 μm, except for the process explained in Section 3.2, where the FEM analysis was used to optimize the cavity diameter. The Young modulus and Poisson ratio used for the SMF in all simulations in this work are typical values found in the literature (73 GPa and 0.17) [11]. The substrate was modeled with the Young modulus and Poisson ratio equal to typical values for ASI 304 steel, namely 200 GPa and 0.28, respectively. The adhesive was simulated by using typical values of Young modulus and Poisson ratio for cyanoacrylate glue Loctite 3400 OptiLOC series, i.e., 3 GPa and 0.31, respectively. The refractive indexes used for the air and the silica were 1 and 1.48, respectively. The photoelastic coefficients used for silica were *p*_11_ = 0.11 and *p*_12_ = 0.25.

**Figure 1:**
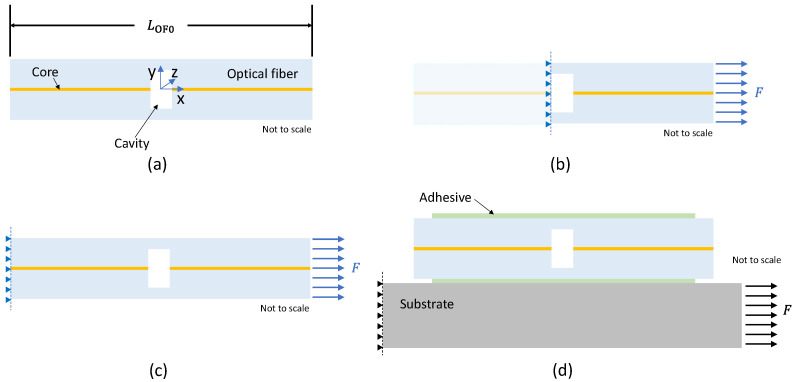
(**a**) Pictorial schematic of the FEM of a single-mode fiber containing an FPI cavity; (**b**) the left side of the cavity was fixed while longitudinal force was applied to the right side of the cavity (Section 2.3); (**c**) in this step, the left side of a fiber section containing the cavity was fixed, while longitudinal force was applied to the right side of the fiber (Section 2.4); (**d**) a substrate was added (gray), and the fiber section containing the cavity was embedded in glue (green). The force was applied to the right side of the substrate, while the left side was fixed (Section 3).

Figure 1 shows a longitudinal cross-sectional cut of the simulated SMF (blue) and FPI cavity (white) when the optical fiber was embedded in glue (green) and attached to the substrate (gray). The center of the coordinate system was placed at the center of the cavity. In the model, the two pieces of SMF and the capillary fiber containing the cavity were modeled as one continuous optical fiber, disregarding the splice.

As mentioned above, the model shown in Figure 1a was first used to simulate the mechanical and optical response of the Fabry–Perot cavity and the optical fiber section with the optical fiber not bonded to the substrate. With this boundary condition, the longitudinal force (X-direction) was initially applied directly to the FPI cavity (Section 2.3) and then applied to an SMF section containing the FPI cavity (Section 2.4 and Section 2.5). In Section 3, the procedure for when the boundary condition was changed to attach the optical fiber to the substrate by means of an adhesive is explained, which is shown in Figure 1d.

### 2.3. Force Applied to the FPI Cavity and Validation of the FEM Model by Comparing with the Theoretical Response

In this section, the FEM model is validated by applying a force directly to the FPI cavity and investigating if it reproduces the expected theoretical behavior for the mechanical deformation, strain, and wavelength shift relation, determined using Equation (5). For this validation, a longitudinal force was applied to the capillary containing the Fabry–Perot cavity of initial diameter and length equal to 75 μm and 20 μm, respectively, and the mechanical deformation and corresponding interference spectra were obtained as a function of applied strain.

**Figure 2:**
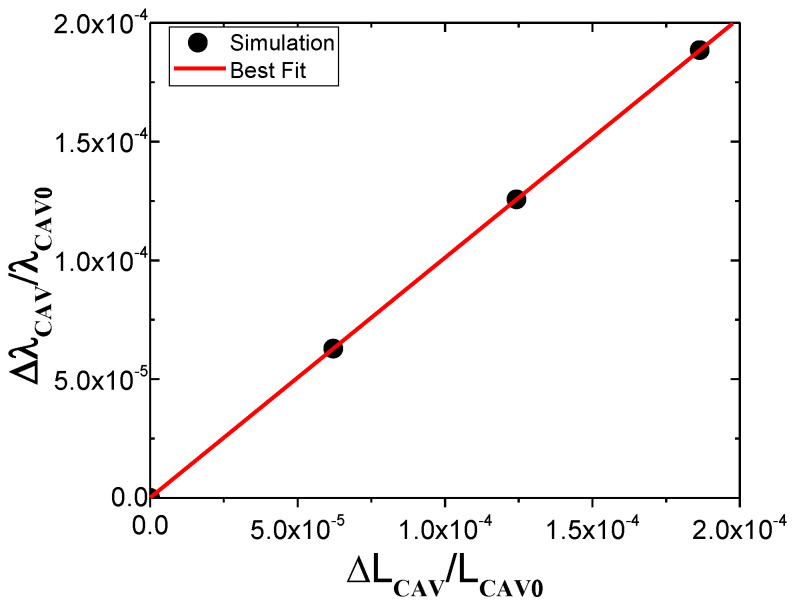
Validation of the FEM by comparing with the theoretical response, determined using Equation (5).

The optical–mechanical relationship obtained from the spectral shift simulated with the model is shown in Figure 2. The solid circles represent the wavelength shift (Δ*λ_CAV_*) normalized by *λ_CAV_*_0_ = 1592 nm for different strain values (*ε_CAV_ =* Δ*L_CAV_/L_CAV_*_0_) applied directly to the FPI cavity. As can be seen in the graph, the angular coefficient of the best linear fit (red solid line) is 1. This result shows that, when the longitudinal strain is applied directly to the cavity, the FE analysis reproduces the theoretical behavior determined with Equation (5) (Δ*λ/λ*_0_ *=* Δ*L/L*_0_), thus validating the model. Similar results were found simulating FPI cavities of other diameters and lengths.

As mentioned earlier, according to Equation (5) and Figure 2, the expected theoretical sensitivity for an FPI cavity with propagating light with a wavelength of *λ_CAV_*_0_ = 1.5 μm, for example, is 1.5 pm/με. However, the experimental results reported in the literature demonstrate higher sensitivities, typically around 10 pm/με [7,18,19], or even 30 pm/με to 40 pm/με and higher [6,11]. The next sections will show that some of these higher sensitivities that have been reported should not be used to determine the absolute strain on the substrate and doing so would give an incorrect measurement. Section 2.4 involves the discussion of the difference between the theoretical sensitivity of a typical FPI cavity and the typical values reported in the literature by investigating the case when a force is applied to the whole optical fiber section, not just to the cavity.

### 2.4. Force Applied to an Optical Fiber Section Containing the FPI Cavity

In this section, we discuss the mechanical deformation, resulting strain, and wavelength shift when a force is applied to the optical fiber section containing the FPI cavity, with the purpose of showing that, in this case, the strain applied to an optical fiber section can be considerably different from the strain experienced by a cavity contained in it [9,11].

#### 2.4.1. Mechanical Deformation

**Figure 3:**
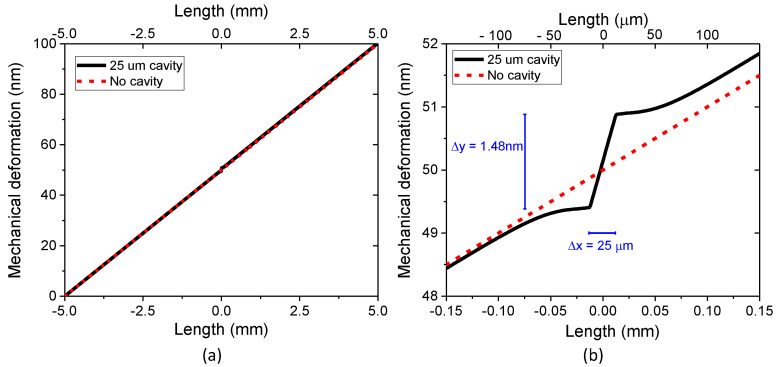
The axial displacement distribution along the center axis of the optical fiber without the cavity (red dotted line) and with the FPI cavity (black solid line) when pulling with 9 mN: (**a**) along the whole length of the optical fiber section (10 mm), and (**b**) zoomed in to a 0.3 mm long section to show the displacement field at the cavity in detail.

To illustrate the difference between the strain applied to the optical fiber section and the resulting strain at the cavity, the proposed FEM model was used to compare the mechanical deformation (elongation) of a 10 mm long section of SMF containing an FPI cavity with a 10 mm long section of SMF not containing an FPI cavity. Figure 3 shows the axial displacement distribution along the center axis of the fiber when pulling one end of the 10 mm section in the X-direction with a longitudinal force (*F*) equal to 9 mN while the other end was fixed. The dashed red line represents the fiber without a cavity, and the solid black line represents the fiber with a Fabry–Perot cavity of diameter = 75 μm and length = 25 μm. Figure 3a shows the mechanical deformation for the whole 10 mm long optical fiber section (*X*-axis from −5 mm to +5 mm), while Figure 3b is a zoomed-in diagram of a 0.3 mm section (*X*-axis from −150 μm to +150 μm).

As expected, for the fiber without the cavity, the force will generate a linear displacement distribution reaching 100 nm at the free end of the fiber, yielding a strain of *ε_OF_ =* Δ*L_OF_/L_OF_*_0_ = 100 nm/10 mm = 10 με along all of the SMF. On the other hand, when the cavity is present, the axial displacement distribution is no longer linear. Since the capillary containing the cavity is composed of less glass than the SMF, and thus is more flexible, it will experience a larger displacement gradient than the rest of the optical fiber. In this case, the optical fiber + cavity will undergo an axial displacement of 100.2 nm at the free end, yielding a total strain of 10.02 με, which is close to the 10 με found for the optical fiber without the cavity. However, as seen in Figure 3b, the 25 μm long cavity will elongate by 1.48 nm, corresponding to an average local strain at the cavity of *ε_CAV_* = Δ*L_CAV_*/*L_CAV_*_0_ = 1.48 nm/25 μm = 59 με. One notices in Figure 3b that, near the interfaces between the optical fiber and the cavity, the slope of the axial displacement field along the fiber’s center axis changes due to the discontinuity in the cross-sectional area, which generates stress concentration and, consequently, also a strain concentration, as visualized with the aid of the strain maps in the next section.

Since in most configurations, the strain measured with this type of sensor is obtained by monitoring the wavelength shifts (Δ*λ_CAV_*) in the FPI interference spectrum, which in turn are a function of the cavity length (Δ*L_CAV_*), the larger elongation undergone by the cavity will yield a measured strain equal to 59 με and not the absolute strain experienced by the optical fiber (10.02 με).

#### 2.4.2. Strain Maps

**Figure 4:**
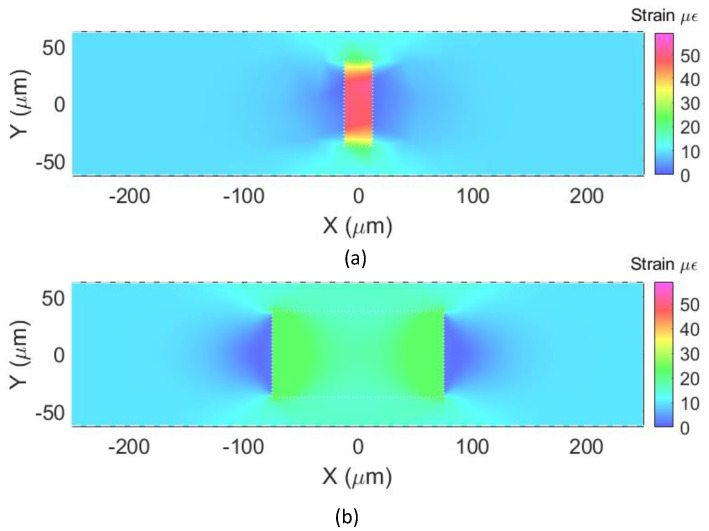
Axial strain maps for a 10 mm SMF section containing an FPI cavity of diameter equal to 75 μm, and length of (**a**) 25 μm and (**b**) 150 μm, being pulled by a longitudinal force of 9 mN.

To illustrate how the nonuniform mechanical deformation experienced along the FPI cavity and the optical fiber affects the resulting strain, in this section, the strain color maps obtained with the FEM analysis are presented. Once again, a longitudinal force (*F*) equal to 9 mN was applied in the X-direction to a 10 mm long section of SMF containing a Fabry–Perot cavity, while the other end was kept fixed. Figure 4 shows an XY-plane cut (Z = 0 and *X*-axis from −250 μm to +250 μm) of the strain maps for two cavities of the same diameter (75 μm), but different lengths, 25 μm (Figure 4a) and 150 μm (Figure 4b).

The maps show that the axial strain in the optical fiber is homogeneous and ε_OF_ ~10 με at a distance of about 100 μm to 250 μm on both sides of the cavity in Figure 4a and at a distance of about 150 μm to 250 μm on both sides of the cavity in Figure 4b. Right next to the vertical walls of the cavity, a decrease in the axial strain of the optical fiber is observed as mentioned in the previous section (Figure 3).

Additionally, as also seen in Figure 3, the apparent strain at the cavities is higher than at the rest of the optical fiber. From the simulations, it can be inferred that the strain at the cavities (*ε_CAV_*) at Y = 0 is ~59 με for the 25 μm long cavity and ~23 με for the 150 μm long cavity. Shorter cavities “concentrate” more strain, experiencing higher local strain. It should also be noted that the simulated strain inside the cavity shown in the maps is only for visualization purposes since they are filled with air.

#### 2.4.3. Wavelength Shifts

**Figure 5:**
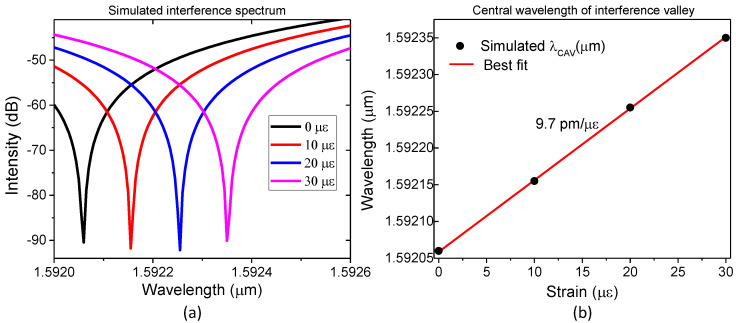
(**a**) FEM simulation of the wavelength shifts in the interference pattern for strain applied to the optical fiber of 0 με, 10 με, 20 με, and 30 με, and cavity diameter and length equal to 75 μm and 20 μm, respectively; (**b**) corresponding central wavelength (*λ_CAV_*) of the interference valley as a function of applied strain.

For typical optical fiber FPI sensors in the literature, the parameter monitored is the wavelength shift (Δ*λ_CAV_*) of the central wavelength (*λ_CAV_*_0_) of a valley or peak in the interference spectrum, obtained from the signal reflected to the input end of the fiber [4,6,9,11]. To illustrate this, the FEM model was adjusted to obtain the mechanical deformation and corresponding interference spectra as a function of the longitudinal strain (ε_OF_) applied to the fiber section containing a cavity. Figure 5a shows the resulting calculated interference spectra produced using a cavity with a diameter and length equal to 75 μm and 20 μm, respectively, for the strain (*ε_OF_*) applied to the fiber section equal to 0 με, 10 με, 20 με, and 30 με. Figure 5b shows the corresponding central wavelength (*λ_CAV_*) of the valley for the same values of applied strain. As can be seen in Figure 5, the interference spectrum shifts linearly to longer wavelengths as the applied strain value increases, as expected.

Calculating the strain sensitivity with the method frequently used in the literature, Δ*λ_CAV_/*Δ*ε_OF_*, where *ε_OF_* is the strain applied to the optical fiber section, will result in a strain sensitivity of approximately 9.7 pm/με. This sensitivity is consistent with typical experimental values reported for optical fiber FPI sensors with similar geometries [4,6,7,18].

### 2.5. Validation of the FEM Model Using Experimental Data

**Figure 6:**
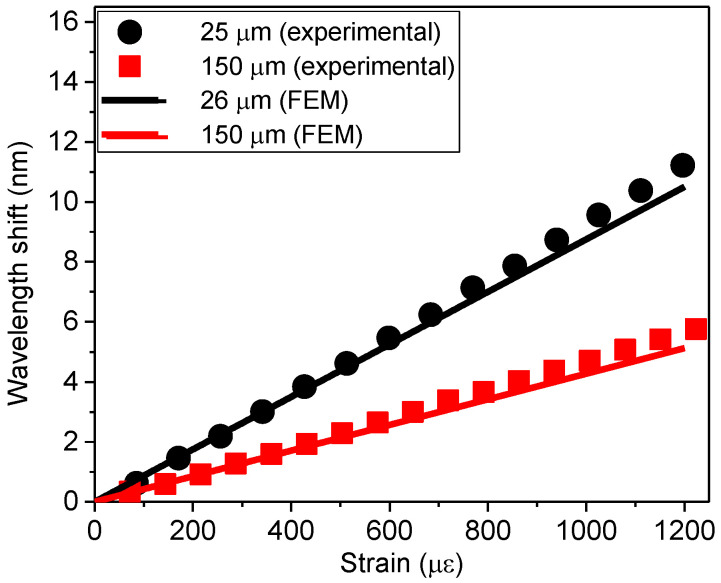
Validation of the developed FEM by comparison with experimental results [4].

Finally, the FEM model was also validated by comparing with experimental data obtained in reference [4] for two FPI cavities (Figure 6). The black circles represent the experimental data for a cavity with a diameter and length of 75 μm and 25 μm, respectively, and the red squares represent the experimental data for a cavity with a diameter and length of 75 μm and 150 μm, respectively [4]. The cavity lengths were experimentally estimated from optical microscope images. The strain sensitivities reported in [4] for these FPI cavities were determined by using the experimental data and the method typically used in the literature, i.e., using the known strain applied to the optical fiber section in the equation sensitivity (*S* = Δ*λ_CAV_/*Δ*ε_OF_*), resulting in a strain of 9.5 pm/με for the 25 μm long cavity and 4.8 pm/με for the 150 μm long cavity.

In the experimental results discussed in [4], the air cavity was obtained by splicing a section of a capillary optical fiber between two pieces of standard optical fiber.

For the validation of the FEM model, the experimental data was adjusted by simulating an SMF optical fiber containing a Fabry–Perot cavity with similar geometry and dimensions, and with a longitudinal force applied to a section of the fiber containing the cavity. The diameter of the FPI cavity was fixed at 75 μm, and the cavity length (*L_CAV_*_0_) was varied to adjust to the best possible fit of the experimental data shown in Figure 6. The results showed that the best fit for the 25 μm long FPI was a simulated cavity with its length and sensitivity equal to 26 μm and 9.5 pm/με, respectively (black line in Figure 6). For the 150 μm long FPI, the best fit was a simulated cavity with its length and sensitivity equal to 150 μm and 4.6 pm/με, respectively (red line in Figure 6). These results are within the uncertainty in the measurement of the cavity length and wavelength shifts for the experimental data; thus, they were considered for the validation of the FEM model.

### 2.6. Force Applied to the FPI Cavity Versus Force Applied to the Optical Fiber

The results in Section 2.3, Section 2.4 and Section 2.5 show that the FPI cavity responds according to theory when a force is applied only to the capillary containing the cavity, but that the cavity and the optical fiber respond differently when a force is applied to the optical fiber section. In this case, the cavity undergoes a higher mechanical deformation than the rest of the optical fiber, as seen in Figure 3. As a consequence, the resulting strain is higher at the cavity than at the rest of the fiber (Figure 3 and Figure 4).

The sensing mechanism of typical optical fiber FPI sensors discussed in the literature relies on changes in the cavity length (Δ*L_CAV_*) to produce wavelength shifts (Δ*λ_CAV_*) of the central wavelength (*λ_CAV_*_0_) of a valley or peak in the interference spectrum. By measuring Δ*λ_CAV_* for a known *λ_CAV_*_0_, one can use Equation (5) to find the applied strain. Alternatively, when the applied strain is known, one can monitor the corresponding Δ*λ_CAV_* to find the sensitivity of the sensor, Δ*λ_CAV_*/Δ*ε_CAV_* (pm/με).

However, because Δ*λ_CAV_* is related to the strain at the cavity, using it in Equation (5) will yield the strain at the cavity, not the strain at the optical fiber. The problem is that, as discussed above, the strain along the rest of the optical fiber section can be considerably different from the strain undergone by a cavity contained in it [9,11]. Consequently, although using *λ_CAV_*_0_ and Δ*λ_CAV_* in Equation (5) will yield the correct strain undergone by the cavity (*ε_CAV_*), the result will most probably be different from the strain undergone by the optical fiber section (*ε_OF_*).

Likewise, the method frequently encountered in the literature to report the sensor sensitivity, i.e., using a known strain applied to the optical fiber section (*ε_OF_*), does not yield the correct sensitivity. This occurs because in most practical lab tests and applications, Δ*ε_OF_* is known, but Δ*ε_CAV_* is not, and Δ*λ_CAV_/*Δ*ε_OF_* and Δ*λ_CAV_/*Δ*ε_CAV_* are usually different. The frequent use of Δ*ε_OF_* instead of Δ*ε_CAV_* to report strain sensitivity would explain why some optical fiber FPI sensors discussed in the literature show strain sensitivities higher than the theoretical result.

So, even though the sensitivities found in Figure 5 and Figure 6 are consistent with typical experimental values reported for optical fiber FPI sensors with similar geometries and are often considered an “enhancement” of the strain sensitivity when compared to the theoretical response, there is no real “enhancement”. In fact, since the local strain at the cavity (Δ*ε_CAV_*) is larger than the strain at the optical fiber (Δ*ε_OF_*), it follows that the real strain sensitivity of the sensor Δ*λ_CAV_/*Δ*ε_CAV_* is smaller than the usually reported Δ*λ_CAV_/*Δ*ε_OF_*. For example, in Figure 5b, if instead of using the strain applied to the optical fiber to find a sensitivity equal to Δ*λ_CAV_/*Δ*ε_OF_*~9.7 pm/με, one had used the actual strain at the cavity (Δ*ε_CAV_*), the strain sensitivity found would have been the theoretical sensitivity, Δ*λ_CAV_/*Δ*ε_CAV_*~1.6 pm/με.

The simulated and experimental results discussed up to now were for cases in which the FPI sensor (optical fiber + cavity) is not bonded to the structure. In the next section, we discuss the relationship between the measurement obtained using an optical fiber FPI strain sensor attached to a structure under strain and the actual strain applied to the structure, i.e., the absolute strain.

## 3. FE Analysis of the FPI Sensor Attached to a Host Structure

### 3.1. FPI Attached to a Structure

As discussed in this section, the FEM analysis was used to investigate the case where the SMF section containing the FPI cavity was bonded on a substrate with an adhesive (Figure 1d). The next sections discuss the obtained results.

#### 3.1.1. Strain

The FEM model was used to obtain the strain profile along the length of the optical fiber and cavity when a force was applied to the substrate. A length of 5.0 mm of the optical fiber was attached on top of the substrate, embedded in the adhesive from x = −2.5 mm to +2.5 mm. The boundary conditions of the tips of the fiber were set to move freely. The boundary condition of one vertical face of the substrate (perpendicular to the *X*-axis) was set to be fixed, while a longitudinal force in the X-direction was applied to the other side, in order to produce *ε_SUB_* = 10 με on the substrate. The fiber and FPI were completely embedded in the adhesive, and the roundness of the optical fiber was not taken into consideration in this FEM model. The substrate was modeled with dimensions equal to 10 mm × 10 mm in the XZ plane, and a height of 2 mm (Y-direction). Other heights were tested from 2 mm to 36 mm, yielding similar results.

**Figure 7:**
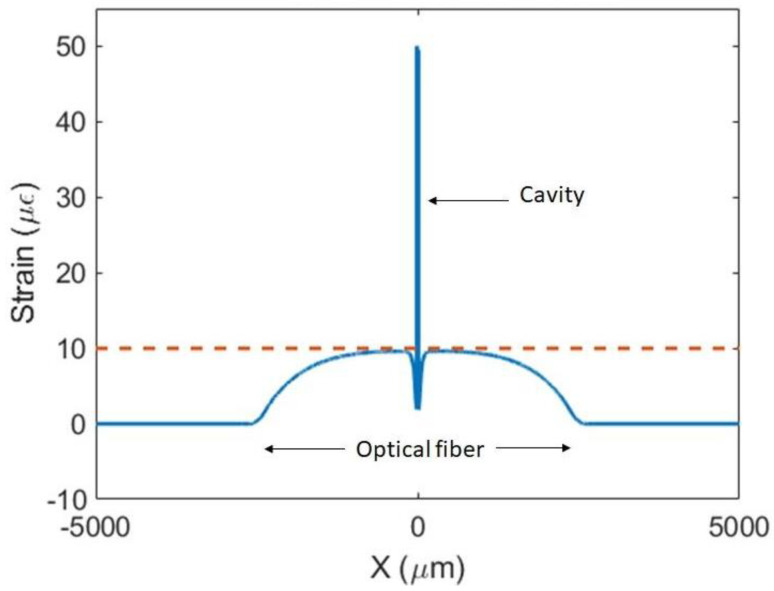
Strain along the center of an SMF with a length of 10 mm, containing a cavity with a diameter of 75 μm and length of 25 μm. Using an adhesive, 5 mm of the optical fiber was attached to the substrate, centered around the cavity. The orange dashed line indicates the 10 με applied to the substrate.

Figure 7 shows the strain along the *X*-axis from −5 mm to +5 mm (for Z = 0 and Y = 0), with the center of the coordinate system placed at the center of the cavity (Figure 1a). As can be seen in the graph, the strain is 0 με along the fiber section not attached and then smoothly increases from 0 με to 10 με along the optical fiber, approaching 10 με near the cavity. However, immediately next to the interface between the fiber and the cavity, a similar effect as seen in the unattached fiber (Figure 3 and Figure 4) is also observed, and a sharp decrease in strain is observed. At the cavity, the strain is approximately 50 με. Other cavity lengths were tested, with similar results.

These results show that this type of FPI-based optical fiber sensor yields the strain at the cavity and not the strain to which the substrate is subjected.

#### 3.1.2. Strain Maps

In this section, we discuss the strain color maps obtained with the FE analysis for the optical fiber and cavities discussed in Figure 4 but now bonded to a substrate by means of an adhesive.

**Figure 8:**
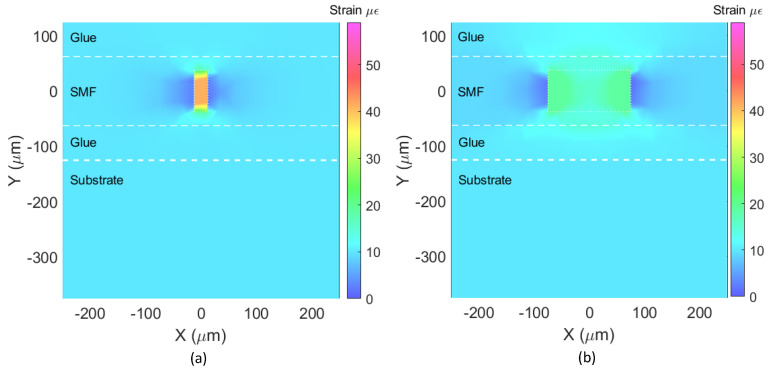
Strain color maps for an SMF section of length of 10 mm containing an FPI cavity of diameter equal to 75 μm, and length of (**a**) 25 μm and (**b**) 150 μm, attached to a substrate that is being pulled by a longitudinal force of 9 mN.

For this case, a longitudinal force of 9 mN was applied to the substrate in the X-direction. It was assumed that the adhesive and bonding were perfect and that the steel block was under an applied strain of *ε_SUB_* = 10 με. Figure 8 shows an XY-plane cut (Z = 0) of the strain maps for the substrate for the *X* axis from −0.25 mm to +0.25 mm, the glue (adhesive), the optical fiber (SMF), and the cavities, for the optical fiber with the 25 μm cavity (Figure 8a) and for the optical fiber with the 150 μm cavity (Figure 8b).

As in Figure 7, the results in Figure 8 show that, along the *X*-axis, the strain approaches 10 με near the cavity but then decreases sharply immediately next to the interface between the fiber and the cavity. Also, the strain at the cavity (*ε_CAV_*) is higher than the strain applied to the host structure (*ε_SUB_*): 42 με at the 25 μm long cavity vs. 19 με at the 150 μm long cavity. As in Figure 4, the simulated strain inside the cavity shown in the strain maps is only for visualization purposes, since they are filled with air.

These results obtained with the FE analysis confirm that the strain value obtained using the FPI sensor is higher than the absolute strain applied to the monitored structure; therefore, the measurement yielded with the sensor should not be directly used to determine the absolute strain on the substrate.

### 3.2. Cavity Optimization for Absolute Strain Sensing of a Substrate

As shown above, typical Fabry–Perot cavities reported in the literature do not yield the same strain measurement as the absolute strain undergone by the substrate, since the cavity elongates more than the rest of the optical fiber. For the same applied longitudinal force (X-direction), cavities with larger diameters and thinner glass walls will stretch more in length and, consequently, produce larger wavelength shifts. To find a cavity geometry that could reduce this effect, mechanical and optical effects were considered in the FEM model to design a Fabry–Perot cavity that yields strain values close to the absolute strain applied to the host structure.

**Figure 9:**
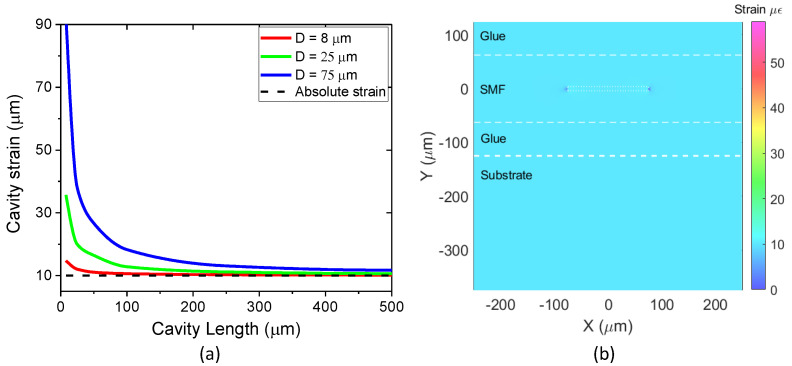
(**a**) Strain measurement using the FPI on SMF containing different cavity geometries; (**b**) strain map for a cavity with a diameter and length equal to 8 μm and 150 μm, respectively.

Figure 9a shows the strain as a function of cavity length (*L_CAV_*_0_ from 0 μm to 500 μm) for three FPI cavities of different diameters (8 μm, 25 μm, and 75 μm). The dashed line in the graph at 10 με represents the strain applied to the substrate. The results show that the strain to which the cavity is subjected approaches the strain applied to the host substrate as the cavity length increases and that cavities with smaller diameters approach this limit faster. As an example of a cavity geometry that yields the correct absolute strain, Figure 9b shows the strain map for a cavity with a diameter and length equal to 8 μm and 150 μm, respectively. As can be seen in the map, the strain at the cavity and the optical fiber match the strain applied to the substrate.

## 4. Conclusions

The finite element method (FEM) was used to model the mechanical and optical responses of an optical fiber Fabry–Perot interferometer (FPI) used as a strain sensor. To validate the model, its results were compared with strain-sensing experimental results presented in [4] and to the theoretical response of the Fabry–Perot cavity.

The model was then used to show that the strain to which the FPI cavity is subjected (*ε_CAV_*) is usually higher than the actual strain applied to the optical fiber (*ε_OF_*), and the substrate (*ε_SUB_*) when the optical fiber is bonded to one. Therefore, while monitoring *λ_CAV_*_0_ and Δ*λ_CAV_* values of an FP cavity will yield the strain undergone by the cavity itself, it will not give the correct strain value applied to the optical fiber section or substrate.

These results provide evidence of the need for calibrating optical fiber Fabry–Perot interferometers used in strain-sensing applications that require the measurement of the absolute strain, at least for most of the FPI geometries that have been proposed in the literature. However, previous calibration might not be feasible for the vast majority of the applications because it would have to be performed by applying a known strain value, and with the optical fiber containing the FPI already attached to the structure to be monitored.

Therefore, for these applications, the FEM model was used to propose a specific cavity geometry that yields the absolute strain experienced by the substrate, without the need for previous calibration of the FPI sensor. Consequently, the proposed FPI geometry can also be a useful tool in the evaluation of the adhesive quality and the bonding of the FPI to the host structure.

## Data Availability

Data underlying the results presented in this paper are not publicly available at this time but may be obtained from the authors upon reasonable request.
